# Interventions for siblings of children and young people with mental health conditions: A systematic review

**DOI:** 10.1002/jcv2.12300

**Published:** 2025-01-23

**Authors:** Irena Tetkovic, Joanna K. Anderson, Danielle Brocklebank, Jessica O’Logbon, Anne‐Marie Burn, Tamsin J. Ford

**Affiliations:** ^1^ Department of Psychiatry University of Cambridge Cambridge UK

**Keywords:** intervention, mental health, siblings, well‐being

## Abstract

**Background:**

Childhood mental health conditions typically affect the entire family, including siblings, however there is a lack of research investigating interventions supporting siblings.

**Methods:**

The review was prospectively registered with PROSPERO (CRD42022377163). We conducted systematic searches of 7 databases (Medline, EMBASE, PsycINFO, Child and Developmental and Adolescent Studies, Applied Social Science Index and Abstracts, Education Research Information Centre, and British Eduction Index) for studies evaluating interventions for children and young people (CYP) with siblings presenting with mental health conditions from January 1980 to August 2024. We included interventions for young people aged 4 to 24 years who have a sibling with a mental health condition, with symptoms and impact lasting at least 3 months. Quality of reporting was assessed using the Critical Appraisal Skills Programme qualitative checklist for qualitative and the Effective Public Health Project Practice quality assessment Tool for quantitative studies.

**Results:**

We identified 4 eligible studies; 2 of family‐based treatment and the other of drop‐in group support from 2469 studies that were screened in total. Quantitative results were rated weak for three out of four studies, while qualitative results were rated moderate for two studies, and high for one study. The two studies of sibling support groups reported high acceptability.

**Conclusion:**

The evidence for the effectiveness of interventions for siblings of CYP with mental health conditions is limited in both size and quality, highlighting a significant gap in research and practice. We cannot draw definitive conclusions from the available evidence, but it suggests support groups have potential to improve sibling outcomes. Additional research is required to determine sibling mental health trajectories and to identify risk and resilience factors possibly influencing poorer mental health outcomes. Clinicians should be mindful of potential effects of mental health conditions on other family members and encourage parents to communicate openly with siblings about family dynamics and the challenges they may face.

**Key points**

- Childhood mental health conditions are challenging for the entire family.
- Practitioners should be aware that mental health problems are more common in children and young people (CYP) who have siblings with mental health conditions.
- Research on interventions for siblings is sparse and of poor quality.
- Practitioners should support parents to speak openly to siblings about family dynamics and their sibling’s difficulties.

## INTRODUCTION

The experience of prolonged mental health conditions in childhood or adolescence presents the entire family system with a challenge, including parents and siblings (Marsh & Johnson, 1997). Young people’s mental health problems may impact their siblings, with reports of stress, distress, and a sense of burden (Shivers et al., 2022). Nearly half (47.5%) of children attending Australian Child and Adolescent Mental Health Services (CAMHS) had at least one sibling presenting with emotional and behavioural difficulties (Ma et al., 2016). Interventions targeting siblings rather than waiting for them to present with mental health conditions could alleviate distress, prevent the onset of mental health conditions and relieve pressure in mental health services.

Two recent systematic reviews demonstrate siblings of children with mental health problems tend to have poorer mental health themselves (Ma et al., 2020) and less warm or more conflictual familial relationships (Ma et al., 2017). These reviews used a broad definition of mental health problems, encompassing diagnosed mental health conditions, elevated scores on behavioural checklists, and past delinquency. Further influences on siblings include stigma (Van Der Sanden et al., 2016), isolation (Stålberg et al., 2004), lower quality of life, poorer family functioning (Barnett & Hunter, 2012), and higher likelihood of using at‐risk coping mechanisms (Kozlowska & Elliott, 2017).

Siblings are in the unfortunate position that unless they too present with a mental health difficulty their needs can often go unacknowledged, even by themselves (McGrath et al., 2024). Therefore, support targeting siblings rather than waiting for them to present with mental health conditions could potentially alleviate distress, prevent the onset of mental health conditions and relieve the pressure on mental health services.

The evidence base for interventions to support siblings of individuals in poor health is sparse, highly heterogenous and largely focussed on long‐term conditions or neurodevelopmental conditions. Although neurodevelopmental disorders may affect siblings in similar ways to mental health conditions (Wolff et al., 2022) and caring responsibilities may seem similar, there is evidence to suggest there are some differences. Wolfe and colleagues’ (2014) longitudinal study found poorer outcomes regarding educational attainment and employability in CYP whose siblings had mental health conditions compared to the general population, whereas this effect was not found for neurodevelopmental conditions. Additionally, there are fundamental differences between the impact of neurodevelopmental and mental health conditions on families. Usually, children present with symptoms of neurodevelopmental conditions in early childhood, whereas mental health conditions typically emerge in adolescence and early adulthood, with peak symptom onset of eating disorder at age 15, for instance, and mood disorders at age 20.5 (Solmi et al., 2022). Furthermore, mental health conditions have a less predictable course and are frequently episodic compared to the persistence of neurodevelopmental conditions (Stein et al., 2020). Consequently, families with mental health conditions need to adjust to these shifts, which may not be the case for parents and younger siblings of children with neurodevelopmental conditions (Taylor et al., 2008).

While previous literature demonstrates a need for interventions for CYP with affected siblings, there is currently no review of existing interventions. There is a need to evaluate existing interventions for CYP with sibling mental health conditions to understand which components of interventions may contribute to their effectiveness. This review aims to address this gap in the literature.

Our objectives were:

1. To synthesise the literature evaluating the impact of interventions aimed at improving well‐being/mental health/quality of life among siblings of CYP with mental health conditions.
2. To synthesise the experiences, beliefs, and perceptions of interventions for siblings of CYP with mental health conditions.

We conducted a systematic review with constructed inclusion and exclusion criteria that we used to extract data to enable us to answer these questions.

For the purpose of this review, mental health conditions are defined as difficulties that last at least three months (Perrin et al., 1993), including internalising conditions such as anxiety, depression, self‐harm, and eating disorders. Siblings with mental health conditions will be referred to as *affected siblings*.

## METHODS

This review was prospectively registered with PROSPERO (International Prospective Register of Systematic Reviews), registration number CRD42022377163 on 13/01/2023 and completed using Prisma guidelines.

### Search strategy and selection criteria

We undertook systematic searches of seven databases for studies published since 1980, available in the English language: MEDLINE and EMBASE via Ovid, PsycINFO, Education Research Information Centre, British Education Index, and Child and Developmental, and Adolescent Studies via EBSCOhost, and Applied Social Science Index and Abstracts via ProQuest. Supplementary search methods included forward and backward citation searching of studies screened in. The search strategy consisted of four elements: terms related to mental health/well‐ being/quality of life, terms related to non‐pharmacological interventions; terms related to siblings, and terms related to mental health conditions (e.g. depression, anxiety, self‐harm, eating disorders). The search strategy used a mix of subject headings (controlled vocabulary) and free‐text terms with subject headings varying by database, depending on terminology used for each database (see Supplementary materials). The last search was performed August 19th 2024.

### Eligibility criteria

We included primary research studies of any design, thereby excluding non‐empirical papers, reviews, systematic reviews or meta‐analyses (to avoid secondary citation and duplication) or philosophical papers, consensus statements, opinion pieces, letters, commentaries, book reviews, discussion or editorial papers. Only published peer‐reviewed papers were included, thereby excluding grey literature.

Table 1 outlines the inclusion criteria (Moher et al., 2010); the main outcome was mental health/well‐being/quality of life in siblings, while secondary outcomes included attitudes/perceived effectiveness/acceptability of interventions of siblings. Studies included mental health interventions not specifically designed to target siblings of CYP with mental health difficulties (e.g. family interventions) if siblings’ mental health was measured as an outcome. Over 75% of the sample must include siblings. Outcomes relating to other family member mental health conditions must be reported separately from sibling outcomes or in a way which enables sibling outcomes to be extracted.

**TABLE 1:** Inclusion criteria.

	Criteria
Population	CYP aged 4–24 years who have a sibling with a diagnosis of a mental health condition, such as depression, anxiety, and eating disorder with symptoms and impact lasting at least 3 months.
Intervention	All psychosocial, non‐pharmacological interventions aimed to support siblings. Family interventions are included if sibling outcome(s) are assessed.
Comparator	Not mandatory but include standard care, no treatment, waitlist controls, and experimental control designs that match for time/contact.
Outcomes	Sibling mental health/well‐being/quality of life reported by child, parent/carer or teacher using a validated scale. Qualitative studies including unaffected siblings’, affected siblings’, parents’/carers’ or clinicians’ experiences and perceptions that improve siblings’ mental health.

The majority of the sample must have a sibling with an internalising mental health condition, such as depression, anxiety, eating disorder or present with self‐harm and the focus of the intervention must not be sibling neurodevelopmental conditions. Internalising mental health conditions constitute the focus of this review due to their increasing prevalence in the UK (Sadler et al., 2018). Although self‐harm is not a mental health condition, it can be seen as an unhelpful coping strategy often associated with poor mental health and extreme psychological distress (McManus et al., 2019).

### Study selection

After compiling the results from all databases and screening for duplicates, titles and abstracts were independently selected by three researchers against our study criteria (IT, DB, JO). Secondly, full‐texts of all publications included in the first stage were screened against the inclusion/exclusion criteria. At both stages, 40% of all records were double screened independently by two researchers (Waffenschmidt et al., 2019). Disagreements were resolved through discussion with the research team.

### Data extraction

The spreadsheet developed for data extraction is available in the supplementary materials (Supplementary Tables 8 & 9), and data extraction was performed by two reviewers (IT and JO). We used the Template for Intervention Description and Replication (TIDieR) reporting checklist (Hoffmann et al., 2014) to extract details of the interventions, including theory of change/rationale, what, where, when and by whom the intervention was provided, adherence to or fidelity of the intervention, tailoring, and modifications.

### Quality appraisal

We used the Critical Appraisal Skills Programme (CASP) qualitative checklist to assess qualitative studies (Long et al., 2020), and the Effective Public Health Project Practice (EPHPP) Quality Assessment Tool for Quantitative Studies (Armijo‐Olivo et al., 2012) due to their suitability to assess the effectiveness of systematic reviews (Deeks et al., 2003). The EPHPP tool evaluates six domains: (a) selection bias, (b) study design, (c) confounders, (d) blinding, (e) data collection, (f) withdrawals/dropouts. These domains are rated as strong, moderate or weak. Scores are averaged to obtain a total score indicating the study’s quality rating.

The CASP qualitative checklist appraises the validity, quality of results based on methodology of the study, and the value of the results contributing to an existing knowledge base and transferability. Mixed methods studies were appraised by both tools Quality appraisals were completed independently by two reviewers (IT and DB) for all included papers. Cohen’s weighted kappa interrater agreement was 0.61 for quantitative papers demonstrating substantial agreement, and 0.49 for qualitative papers demonstrating moderate agreement. Disagreements were resolved via discussions.

### Synthesis

Sparse qualitative findings prevented a thematic synthesis (Popay et al., 2006), thus findings are presented in narrative form.

## RESULTS

**FIGURE 1:**
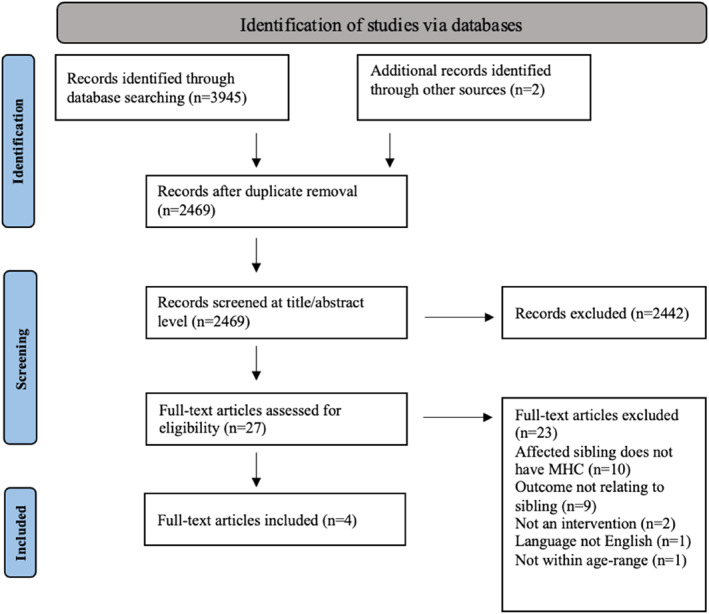
Flow diagram depicting selection process. *Note: MHC stands for mental health condition.

The initial search generated 3945 publications, of which 2469 remained after duplicates were removed (Figure 1) for article title and abstract screening. Two additional records were identified through forward searching by searching for studies that cited the published papers that met our criteria. In total, 27 full texts were screened for eligibility, of which 4 were included in the final review. Two studies (Feriante et al., 2022; Rubin et al., 2018) included due to their qualitative aspects, also collected quantitative data using non‐validated measures that did not fit our inclusion criteria. Similarly, one potential quantitative study (Foster, 2016) was excluded as the intervention did not address support for CYP with sibling mental ill health specifically, and most of the sample had an affected parent (*n* = 43) rather than an affected sibling (*n* = 11). This study will not be reported in the results, but more details are provided in the supplementary material.

### Study characteristics

All included studies were conducted in the past decade and in Australia (Van Langenberg et al., 2016, 2018) and the USA (see Table 2; Rubin et al., 2018; Feriante et al., 2022). One study used a cohort design (Van Langenberg et al., 2016), another study utilised semi‐structured interviews (Van Langenberg et al., 2018), while two studies used surveys (Feriante et al., 2022; Rubin et al., 2018). Two research groups conducted two studies each; a pilot and a continuation of the sibling support group (Rubin et al., 2018 & Feriante et al., 2022) and two analyses of family‐based treatment (FBT), initially with mainly quantitative scores and subsequently in comparison with parent‐focused treatment (PFT) (Van Langenberg et al., 2016 & Van Langenberg et al., 2018). More details about the interventions offered are provided in Table 2, while Table 3 describes the research methods applied.

**TABLE 2:** Description of interventions included.

First author, year	Rationale/theory of change	What	Who provided	Where	When and how much	Tailoring	Modifications	How well
Van Langenberg, 2016	Family seen as resource to support recovery of affected sibling	FBT treatment for eating disorder (Lock & Le Grange, 2013): ‐ Acknowledging siblings as taking up parental role and giving them less responsibility ‐ Acknowledging parental and sibling guilt ‐ Reinforcing healthy intergenerational boundaries between sibling subsystem and parental subsystem	Trained mental health clinicians under supervision of the third author*	Eating disorders programme out‐patient clinic, in Australia	18 sessions, over approximately 6 months in‐person with the family and clinician for each family individually	N/A	N/A	N/A
Van Langenberg, 2018	Core principles behind PFT are the same as in FBT (see above), however treatment excludes siblings. PFT & conjoint FBT both adhere to the treatment manual (Lock & Le Grange, 2013)	Conjoint FBT (see above) and PFT where parents attend support during ill child’s treatment but no provision for siblings. PFT: ‐ Focus and content of PFT matches conjoint FBT, however parents and ill child treated separately.	Mental health clinicians for PFT and FBT	Multidisciplinary specialist outpatient based eating disorder service, in Australia.	Both conjoint FBT and PFT ∼18 sessions, over 6 months	N/A	N/A	N/A
Rubin, 2018 (pilot)	Peer mentoring aiming to: Increase resiliency & reduce trauma of sibling’s psychiatric hospitalisation; Skill, competency, and confidence devleopment in parents; Restoration of family stability to minimise re‐hospitalisation; Provide opportunity for siblings to connect with other siblings	Caregivers’ brochure: “Supporting siblings of children with mental health needs” ‐Caregiver psycho‐educational group: Covered needs of siblings, importance of and strategies to support siblings. Sibling support group activities (see below)	Sibling support group provided by clinical mental health trainees, with an expressive art therapist & a social worker. Psychoeducational caregiver group provided by a trained parent mentor	CHA cambridge hospital, in cambridge, Massachusetts, US	‐ 1.5 h bi‐weekly “drop‐in” during and after patient’s hospitalisation. ‐ 12 siblings per group for siblings and for caregivers‐participants per in‐person group at same time ‐ 97% caregivers & 92% siblings attended only one session	Sibling group was tailored for age of attendees	‐ Modified recruitment method for intervention: From flyer distribution by medical team & calls by child and adolescent to calls made by trained parent mentors	‐ Potential lack of fidelity: Modified group activitity based on group size, composition, and timing restrictions ‐ Aims to evaluate reduction in sibling’s trauma, increase resiliency, remission rates, and restoration of family stability after affected sibling’s return home not addressed in paper
Feriante, 2022	‐ Theory of change: Dr. Bowen’s family systems theory (Bowen, 1978). Objectives: ‐ Peer support for all family members, ‐ Provide space to share experiences in safe and understanding enviornment ‐ Parent mentors incorporated into intervention to empower and educate parents/caregivers Aims: ‐ Improve sibling’s and caregivers’ knowledge of coping skills ‐ Influence their intent to use supportive skills and resources in family	Sibling support programme: Family‐centered mental health intitiative providing peer support, parent mentor guidance, clinical‐led group therapy (see above) Caregiver psychoeducational groups for first‐time participants encourage parent‐to‐parent support ‐ Structure: Building group as safe place, Informing caregivers about parent mentor facilitator gives information about sibling experience, parenting strategies promoting resiliency and reducing trauma, and relevant resources, Caregivers share stories and provide support to each other	Co‐facilitation of sibling group by mental health trainees. Caregiver groups facilitated by trained parent mentor, both groups supervised by clinical social worker	At inpatient child and adolescent psychiatry units, outpatient clinical, and community centres, in the US	‐ Groups at inpatient units offered weekly, at community sites sessions offered monthly ‐ Sibling group intitially in‐person,during covid‐19 pandemic groups moved online	N/A	‐ During covid went from in‐person to online	N/A

*Note*: Not specified whether qualified practitioners or researchers trained to provide just this treatment.

Abbreviations: CHA, Cambridge health alliance; FBT, family‐based treatment; PFT, parent‐based treatment.

**TABLE 3:** Description of studies and findings.

First author, year	Intervention	Sample size and demographic characteristics	Outcome measures (indicate measures used and whether self‐, parent‐, carer‐ or clinician‐reported)	1. Mental health/well‐being/quality of life	2. Attitudes/perceived effectiveness/acceptability	Comments
Van Langenberg, 2016	FBT for anorexia nervosa	‐ 46 siblings (56.4% female, mean age = 16.4 (SD = 4.3)) ‐ Affected siblings: 80 (90%) female, mean age = 15.1 years (SD = 1.8), mean duration of mental health condition = 9.2 months (SD = 6.8)	‐ Strengths and difficulties questionnaire (SDQ; Goodman, 2001) as a measure of mental health, self‐reported and parent‐reported pre‐ and post‐treatment	‐ No sign. Difference in sibling psychosocial well‐being pre‐ and post‐treatment, measured by SDQ	‐ *Expectations and perceptions of sibling’s wellbeing: No sign. difference* ‐ *Most reported no effect after treatment* ‐ *1/3 siblings, affected siblings, fathers, and mothers indicated negative effect on sibling*	‐ No information on SES of participants Pre‐treatment: ‐ Increased psychological distress on SDQ compared to population norm pre‐ and post‐treatment (sibling‐ and mother‐reported) ‐ Utility of comparison against norms post‐treatment less useful ‐ Sample probably lacks power & likelihood of spurious positive finding high as multiple testing by both parents, sibling, comparison group across sub‐scales of SDQ
Van Langenberg, 2018	FBT for anorexia nervosa	‐ n = 12 siblings (10 female, ages 10–18, mean age = 14.6 (SD = 2.9). ‐ Affected siblings’ conditions was anorexia nervosa ‐ 7 patients and 14 parents ‐ 7 siblings in FBT condition, 5 siblings in PFT condition	Semi‐structured interviewing: ‐ Five themes extracted from sibling interviews. ‐ Four themes in parent and affected sibling interviews. ‐ Interview topics: Sibling involvement and experience of treatment; sibling role at home during treatment; quality of sibling relationship before, during, and after treatment; impact of ED and treatment on sibling; potential changes regarding affected sibling	‐ Sibling experiences less secret worry about affected sibling and space to communicate own feelings (parent‐reported)	‐ Sibling participation in FBT declined over time due to not feeling part of treatment, boredom, other commitments (sibling‐reported) ‐ Sibling uncertainty about purpose of attendance ‐ Parents no clear preference for attending ‐ Pre‐treatment most siblings took on multiple roles, that is, household duties, parent or affected sibling support (sibling‐reported) ‐ Siblings post‐treatment in conjoint FBT: Less likely to take on parental responsibilities (sibling‐, affected sibling‐, and parent‐reported)	‐ Sibling age: No age‐range provided. Contacted authors for confirmation, however mean age is within range, SD suggests that if participants’ age>24, will likely be a very small fraction ‐ No information provided on SES of participants ‐ 3/5 siblings in PFT: Feeling excluded, limited access to information about disorder and treatment, lacking knowledge to contribute (sibling‐ reported)
Feriante, 2022	Sibling support programme: Family‐centered mental health initiative	‐ Siblings aged 6–18 ‐ *n* = 505 total, *n* = 455 in‐person, *n* = 50 virtual	‐ Survey about knowledge gain, satisfaction, and perceived change, with categorical answers (sibling‐reported and caregiver‐reported) ‐ Qualitative perceived change pre‐ and post‐treatment (sibling‐reported and caregiver‐reported)	‐ Approximately half of siblings felt negative pre‐treatment ‐ Majority of siblings felt positive post‐treatment	‐ *Majority of siblings enjoyed recognising and articulating shared experience* ‐ *Majority of siblings would recommend group* ‐ *Majority of caregivers enjoyed attending group and would recommend*	‐ Qualitative impressions were coded into three categories: Positive, neutral, and negative. Categorisation method used for outcome was not explained. ‐ Demographic information collected mostly from community site, not for all participants involved. ‐ Post‐intervention surveys completed about expectations before intervention. ‐ Survey feedback collected only after initial session ‐ Siblings: 266 (53%) female, 168 (33%) 5–9 years, 246 (49%) 10–14 years, 87 (17%) 15–19 years
Rubin, 2018	Sibling support demonstration project	‐ *n* = 145 siblings (49% male, 51% female), 196 caregivers ‐ Family member admitted to psychiatric in‐patient unit ‐ Siblings aged 5–19 growing up in same household	‐ Sibling qualitative data during Starburst Candy Activitiy and after asking open‐ended survey questions. Youngest participants’ feedback sometimes via drawings, which were translated to words by group leaders ‐ Caregivers quantitative questionnaire: 5 options measuring: satisfaction, group helpfulness, level of comfort sharing in group, and whether they would recommend to other families	N/A	‐ Many siblings felt comfortable and upset to leave group, ‐ Most siblings relieved post‐session: Liked sharing experience ‐ Many siblings describing apprehensions before session ‐ Most helpful for siblings: Being able to discuss and ask questions ‐ *Most siblings would recommend group to others* ‐ *Many siblings reported psychoeducation helpful*	‐ Themes identified through grounded thematic analysis ‐ Anecdotal evidence of siblings who would not recommend group suggests may be due to misunderstanding question ‐ Siblings age: −39.3% = age 12–18, 34.3% = age 9–11, 26.4% = age 5–8

*Note*: Sibling conditions included: Attention‐deficit/ hyperactivity disorder (ADHD), disruptive mood dysregulation disorder (DMDD), oppositional defiance disorder (ODD), reactive attachment disorder (RAD), Borderline Personality Disorder (BPD), Autism, Anxiety and Depression (confirmed by author via email).

Abbreviations: CP, conduct problems; ED, emotional difficulties; FBT, family‐based treatment; H/I, hyperactivity/impulsivity; PFT, parent‐based treatment; PP, prosocial problems; sign., significant; SD, standard deviation; SES, socio‐economic status; TD, total difficulties.

Findings in italics symbolise quantitative findings from bespoke scale in studies that have been included due to their qualitative aspects and would not otherwise be included in this systematic review based solely on their quantitative results.

The number of participants ranged from 12 to 505 siblings and comprised 708 siblings in total.

Mean participant age ranged from 14.6 to 16.3 years, where reported (Van Langenberg et al., 2016, 2018).

One pair of studies were conducted in outpatient eating disorder clinics (Van Langenberg et al., 2016, 2018), while one was conducted in the paediatric psychiatry department of a hospital (Rubin et al., 2018), expanded to multiple sites, including an inpatient child and adolescent psychiatry unit, outpatient clinical setting, and community centres (Feriante et al., 2022).

The level of detail about interventions was assessed using TIDieR criteria (i.e. intervention reason, intervention location, location deliverer; Hoffmann et al., 2014).

Missing elements included fidelity and demographic information. Demographic information, such as socio‐economic status (SES),—a well‐established moderator of mental health outcomes (Lawson & Mace, 2010)— was missing or incomplete in three of the four studies (Feriante et al., 2022; Van Langenberg et al., 2016; Van Langenberg et al., 2018), the age range of participants was missing in one study (Van Langenberg et al., 2018), and age and gender were missing for most non‐community participants (Feriante et al., 2022).

### Measures

Each of the four studies used different outcome measures. Although some of the studies used quantitative methods and are discussed here (Feriante et al., 2022; Rubin et al., 2018), their quantitative outcome measures did not meet our inclusion criteria as they utilised unvalidated scales; thus only qualitative results from those studies are formally included. Unvalidated scales were excluded from our work as they could introduce potential bias or error which might confound results.

#### Family‐based treatment

Van Langenberg and colleagues’ (2016) study used the Strength and Difficulties Questionnaire (SDQ) comparing pre‐and post‐treatment scores of siblings and then comparing these with age‐adjusted population norms (Goodman, 2001). Additionally, they used an unvalidated, bespoke Likert‐scale‐based measurement of treatment expectations and impressions on sibling well‐being. Feriante and colleagues’ (2022) study employed post‐intervention surveys that explored expectations prior attending the group as well as post‐group impressions.

#### Sibling support group

In comparison, Rubin and colleagues’ (2018) approach included open‐ended questions during the programme which were analysed using grounded thematic analysis. Caregivers completed a bespoke questionnaire to rate their impressions of the usefulness of the programme.

#### Family‐based treatment/parent‐focused treatment

Van Langenberg et al. (2018) conducted semi‐structured interviews with families who underwent FBT and PFT, exploring sibling involvement and experience of treatment, their roles at home, quality of the sibling relationship before, during, and after treatment, and the impact of the eating disorders and treatment on the sibling. Analysis generated themes relating to FBT and PFT.

### Interventions (see Table 2)

The FBT approach to eating disorders offers in‐person treatment for young people with eating disorders across 18 sessions over 6 months (Van Langenberg et al., 2016, 2018) alongside family members, including unaffected siblings. Parent‐focused treatment is offered for affected siblings and parents separately and does not include unaffected siblings.

The intervention aims to provide a space for siblings to learn more about the affected sibling’s condition. Family‐based treatment actively encourages siblings to emotionally support the affected sibling and explicitly acknowledges parental and sibling guilt. Recognising that siblings typically take on a caregiver role, this programme aims to enforce intergenerational boundaries, whereby siblings are not expected to take on parental responsibilities. Content taught in PFT aligns with FBT.

Family‐based treatment was delivered by a mental health clinician for family members, whereas PFT was performed by nurses for ill siblings and mental health clinicians for parents. Level of adherence to protocol was not described, thus we cannot draw any conclusions about fidelity.

The sibling support group (Feriante et al., 2022; Rubin et al., 2018) also aims to create an environment where siblings can gain a better understanding of their affected sibling’s condition. The intervention further strives to ensure siblings feel included in the conversation about the affected sibling. The support group provided support for unaffected siblings whose affected sibling’s mental health condition warranted hospitalisation (Rubin et al., 2018), and included a drop‐in psychoeducational group separately for siblings and for caregivers. While the pilot study (Rubin et al., 2018) was delivered by psychiatry residents weekly in a hospital setting face‐to‐face, the continuation of the siblings support programme (Feriante et al., 2022) also included face‐to‐face monthly in‐person community sessions as well as online drop‐in sessions in both hospital and community setting following the covid‐19 pandemic.

Most siblings and caregivers only attended one support group session. During the group, siblings completed activities to provide them with the opportunity to connect with others with shared experiences, to gain greater knowledge of the affected sibling’s condition, and to develop healthy coping strategies. The caregiver psychoeducational group utilised trained parent mentors with lived experience to increase credibility with other caregivers, while the curriculum for the sibling support group was adapted from SibShop—a programme aimed at sibling of children with disabilities (Meyer & Vadasy, 2008). The pilot support group (Rubin et al., 2018) also aimed to accommodate to different age ranges and to offer flexibility according to attendance that varied by sessions, while content remained similar. Neither of the sibling support group studies (Feriante et al., 2022; Rubin et al., 2018) reported the range of sessions participants attended, nor did they enquire reasons for attendance of group, however it would be useful to know the characteristics of the 8% who attended more than one session in the pilot (Rubin et al., 2018) and to explore why they chose to do so. Adherence to protocol of the continuation of the programme (Feriante et al., 2022) was not described.

### Quality of included studies

The quality of included studies was weak for quantitative approaches formally in the systematic review (See Supplementary Figure 1; Van Langenberg et al., 2016), and poor for those using bespoke scales, which are out of the scope of this systematic review (See Supplementary Figure 1; Rubin et al., 2018; Feriante et al., 2022). Weaknesses included selection bias due to failures to report how many eligible participants declined to participate and lack of control of confounders by not considering the potential impact of demographic factors such as SES. Further weaknesses include lack of information when describing adherence to protocol by all four included studies as well as information provided about session attendance being insufficient (Feriante et al., 2022; Rubin et al., 2018) and not available (Van Langenberg et al., 2016, 2018).

The quality of included qualitative work was moderate (Feriante et al., 2022; Rubin et al., 2018) and high (Van Langenberg et al., 2018). Shortcomings include not exploring themes in detail (Feriante et al., 2022; Rubin et al., 2018), drawing quantitative inferences from qualitative data without explaining the process behind these inferences (Feriante et al., 2022) and collecting impressions of the intervention post‐treatment and comparing them to retrospective reports of expectations of the group (Rubin et al., 2018), thereby introducing information bias to the data.

The measures used to assess siblings varied substantially between studies. Ideally, studies would collect measures relating to mental health pre‐and post‐treatment using a validated scale and compare outcomes to a control group. For qualitative studies, participants' experiences and attitudes of interventions would be presented with a clear development of themes from a described interview topic guide. Furthermore, studies would ideally monitor the adherence to protocol and participant session attendance.

In contrast, the included studies largely failed to implement validated scales with pre‐ and post‐ measures and seldom used control groups for quantitative aspects, while rarely reporting on fidelity and participant session attendance, and seldom presented qualitative findings clearly.

### Findings

#### Mental health/well‐being/quality of life

The impact of interventions on mental health, well‐being or quality of life was only assessed using a validated scale by one study (Van Langenberg et al., 2016; see Table 3) and showed no significant difference in sibling psychosocial well‐being pre‐ and post‐treatment, as measured by the SDQ.

The perceived impact of interventions on mental health, well‐being or quality of life was assessed qualitatively by two studies (Feriante et al., 2022; Van Langenberg et al., 2018). In Van Langenberg and colleague’s study (2018) some parents reported perceptions of siblings experiencing less secret worry over the affected sibling and having more space to communicate their feelings while Feriante et al. (2022) reported increased positive feelings in siblings post‐treatment compared to pre‐treatment.

#### Qualitative experience and acceptability

The experience and acceptability of interventions was assessed qualitatively by two studies (Rubin et al., 2018; Van Langenberg et al., 2018). In Van Langenberg and colleagues’ study (2018) siblings reported uncertainty about the purpose of their attendance in FBT. The latter was attributed to feeling excluded from the treatment process, boredom, or other commitments. Parents had no clear preference about siblings attending the group, although some reported concerns over sibling attendance because they were concerned that siblings may copy the eating disorder behaviour. Post‐treatment, sibling involvement in the household was mixed, with some unaffected siblings only providing emotional support to their affected siblings while others also took on active caregiver roles, based on reports by affected siblings and parents. This suggests FBT may have helped some siblings formulate healthy boundaries, as FBT usually leads to tension between leveraging support from siblings while also making sure they do not take on too much responsibility. However, it seems these boundaries were not communicated clearly enough for all participants. Siblings who were not directly involved in treatment because their parents undertook PFT described feelings of exclusion and of limited access to information regarding disorder and treatment. In contrast, siblings in FBT were directly involved and reported an increase in their knowledge and understanding of their affected sibling’s condition and their sibling’s experience.

In Rubin and colleague’s (2018) sibling support group, siblings reported experiencing greater emotional support and comfort through the intervention as well as greater knowledge about their sibling’s condition and coping strategies. Some siblings said the group was a supportive environment and “safe place” which they felt “sad” and “scared” to leave. Thus, both sibling support group and FBT approaches resulted in greater self‐reported depth in knowledge.

## DISCUSSION

We reviewed the evidence‐base for interventions aimed at protecting the mental health of siblings of CYP with mental health conditions; Our findings suggest that the area is under‐researched and studies conducted to date have severe limitations. Only four studies met our criteria, of which two assessed FBT, an intervention that was not specifically aimed at improving siblings’ mental health, and in fact might undermine it (Van Langenberg et al., 2016, 2018). Due to the small number of studies, methodological heterogeneity, weak methodology overall, and variety in outcome measures, it is impossible to draw firm conclusions on the effectiveness of interventions for CYP whose siblings present with mental health conditions. However, sibling support groups seem acceptable, suggesting a single drop‐in session might be sufficient, even if the affected sibling suffers from difficulties severe enough to warrant admission. In contrast, FBT was not associated with improvements in well‐being and even elicited perceived negative effects on sibling well‐being as indicated by approximately a third of siblings, affected siblings, and parents (Van Langenberg et al., 2016). Future research requires more robust methods, including longer follow‐up period.

Quantitative findings from studies that did not meet inclusion criteria were not reported, yet merit consideration of experience or acceptability of interventions (Feriante et al., 2022; Rubin et al., 2018; Van Langenberg et al., 2018). Feriante and colleagues’ (2022) drop‐in group suggests positive outcomes for siblings related to increased perceived knowledge of coping skills, opportunity for respite, and the ability to articulate shared experience. Both Rubin and colleagues’ (2018) and Feriante and colleagues’ (2022) work suggests acceptability of their drop‐in groups by siblings and caretakers, while Van Langenberg et al. (2018) found declining attendance of siblings over time, suggesting either decreasing acceptability over time or that fewer sessions sufficed. The declining attendance suggests that moving towards a shorter intervention design might benefit participants, although further information regarding reasons for non‐attendance would be needed to ensure evidence‐based implementation.

Our findings are mirrored in the wider sibling support literature, as a systematic review of interventions for siblings of children or young people with neurodevelopmental conditions (Wolff et al., 2023) similarly reported great methodological variation and weakness. The aims of the interventions described are comparable to those for CYP with siblings with neurodevelopmental conditions, as these interventions typically employ psychoeducational and psychosocial elements to improve understanding of sibling’s condition and teach adaptive skills (Wolff et al., 2023). In terms of the scope, Wolff and colleagues’ (2023) systematic review also investigated sibling mental health and well‐being outcomes following psychosocial interventions in CYP. Unlike our study, they only included self‐reported qualitative sibling outcomes and they also examined risk and protective factors associated with outcomes. Our study did not find any self‐reported improvements in sibling mental health or well‐being apart from relief post‐intervention (Rubin et al., 2018), as did the recently updated systematic review of interventions for CYP with siblings with neurodevelopmental conditions (Wolff et al., 2023). The latter also indicated the importance of parent/caregiver involvement, and notably all four included studies in the current review involved parents/caregivers.

In contrast, five previous systematic reviews of sibling neurodevelopmental conditions did find improvements in unaffected sibling self‐reported anxiety, depression and quality of life (Hartling et al., 2014; Kirchhofer et al., 2022; McKenzie Smith et al., 2018; Thomas et al., 2016; Tudor & Lerner, 2015). This discrepancy might be explained by Wolff and colleagues’ (2023) integration of 13 additional studies of better quality.

In line with past literature on interventions for siblings of CYP with neurodevelopmental conditions (Hartling et al., 2014; Kirchhofer et al., 2022; McKenzie Smith et al., 2018; Thomas et al., 2016; Tudor & Lerner, 2015) we found increases in self‐reported knowledge about the sibling's condition in FBT (Van Langenberg et al., 2018) and the sibling support group (Rubin et al., 2018). A review of interventions for mainly adults—with only one intervention for individuals aged 16–60 — with sibling mental health conditions (Acri et al., 2017) suggests peer‐delivered programmes may be beneficial. Notably, it is unclear whether this translates to CYP, and which format of intervention may benefit siblings most. Our systematic review suggests that caregiver groups delivered by peers are beneficial.

### Strengths and limitations of the study

To our knowledge, this is the first systematic evaluation of interventions for CYP whose siblings present with mental health conditions other than neurodevelopmental disorders; an often overlooked population. We used robust methodology, including searching multiple databases, double screening and data extraction and active supplementary search strategies. Our conclusions are restricted by the small number of studies as well as their methodological limitations. We only included peer‐reviewed studies, thereby excluding grey literature, which might include potentially effective interventions that might warrant future investigation. Given the poor quality of peer‐reviewed literature already included in our review, it is unlikely these interventions would have contributed to achieving a clearer understanding.

However, due to the small number of peer‐reviewed work it is possible future work could potentially benefit from drawing on the wider literature. The inclusion of grey literature would need to be assessed with the same attention to quality as conducted in this and most systematic reviews.

### Implications for future research and practice

Poorer mental health and frequent presentation to clinical services for siblings suggest the need for support, but we lack data on who is most at risk of adverse outcomes, and how best to deliver effective support. A study which we excluded from our review that investigated a respite programme for CYP mostly caring for parents (*n* = 22) also included two CYP with caring responsibilities (Wind & Jorgensen, 2019). Quotes from these two siblings suggest their appreciation for being given space in their own right and therefore not wanting to use this space to discuss their affected sibling as well appreciation for the ability to share information they did not feel comfortable discussing with caregivers. This suggests a desire for support which some siblings communicate in other studies (Feriante et al., 2022; Rubin et al., 2018; Sangha et al., 2023; Van Langenberg et al., 2018) that needs further exploration, in addition to the types of support seems best.

Future studies should use validated measures or established qualitative approaches, ideally with a standardisation of outcome measures and methods to support a more meaningful synthesis of results. Qualitative research will provide valuable insights regarding the acceptability and feasibility of interventions.

Further epidemiological research is needed to establish mental health trajectories for siblings and to understand empirically which risk and resilience factors may impact poorer mental health outcomes, as well as to estimate how many siblings develop difficulties that require clinical treatment. Further research might also benefit from investigating the impact of interventions on well‐being outcomes that are not commonly addressed. Examples include comparing the outcomes of interventions with and without parent/caregiver participation or Foster and colleagues’ (2016) investigation of the effects of their intervention for family mental health conditions on unaffected siblings’ hope and connections within and outside of their family. We need to know which characteristics may influence siblings’ outcomes in general and especially following interventions. Our current lack of knowledge in relation to characteristics is demonstrated by our mixed findings and previous literature in this area (Wolff et al., 2023). Certain characteristics, such as unaffected sibling age, affected sibling’s mental health condition type, severity, and duration, and overall sibling well‐being pre‐treatment seem important potential predictors to explore. Wolff and colleagues' (2023) paper suggests that siblings with lower baseline self‐esteem and coping skills benefitted most from interventions for siblings in relation to neurodevelopmental conditions.

We recommend that future intervention studies also link with administrative data to enable long‐term follow‐up of outcomes, extending across different life phases. We define long‐term outcomes as extending beyond 5 years.

In terms of current clinical practice, practitioners need to consider the potential impact of mental health conditions on other family members and support parents to speak openly to siblings about the family dynamics and potential difficulties they may experience while their sibling is struggling. With the current pressures on CAMHS, there is less of a focus on working with parents and families (Faulconbridge et al., 2015) that might serve to undermine recovery, and thus additional sessions including family members or drop‐in sessions for parents or siblings might be cost‐effective in preventing relapse or additional referrals.

Practitioners should be aware of two ethical implications when including unaffected siblings in treatment. First, there is a potential risk of diverting resources away from the index patient. Therefore, there is a need to balance the focus between the affected individual and their sibling to ensure that the care of the primary patient is not compromised. Second, including unaffected siblings in treatment must ensure the benefit outweighs the risk. Thus, continuous evaluations are needed to track the unaffected sibling’s well‐being.

Given these ethical implications and current evidence‐base, services are advised to provide information and resources to unaffected siblings and support parents in monitoring the unaffected sibling’s well‐being, offering intervention only if indicated.

## CONCLUSION

Prior research (Ma et al., 2017) demonstrates that CYP with sibling mental health conditions struggle more than the average population with their own mental health. The four studies evaluating interventions for CYP with sibling mental health conditions suggest that the literature is small and of poor quality. Sibling support groups may offer relief to siblings, however these outcomes have hitherto not been measured robustly.

## AUTHOR CONTRIBUTIONS

**Irena Tetkovic**: Conceptualization; formal analysis; methodology; writing—original draft; writing—review & editing. **Joanna Anderson**: Methodology; supervision; writing—review & editing. **Danielle Brocklebank**: Formal analysis; writing—review & editing. **Jessica O’Logbon**: Formal analysis; writing—review & editing. **Anne‐Marie Burn**: Methodology; supervision; writing—review & editing. **Tamsin J Ford**: Conceptualization; methodology; supervision; writing—review & editing.

## CONFLICT OF INTEREST STATEMENT

T.F.‘s research group receives funding from Place2Be for research methods consultancy.

## ETHICS STATEMENT

No ethical approval or patient consent was required for this research review.

Goodyear et al., 2009; Laurent et al., 1999; Snyder et al., 1997.

## Supporting information

Supplementary material

## Data Availability

The data that support the findings of this study are available from the corresponding author upon reasonable request.

## References

[jcv212300-bib-0001] Acri, M. , Hooley, C. D. , Richardson, N. , & Moaba, L. B. (2017). Peer models in mental health for caregivers and families. Community Mental Health Journal, 53(2), 241–249. 10.1007/s10597-016-0040-4 27344658 PMC5555254

[jcv212300-bib-0002] Armijo‐Olivo, S. , Stiles, C. R. , Hagen, N. A. , Biondo, P. D. , & Cummings, G. G. (2012). Assessment of study quality for systematic reviews: A comparison of the cochrane collaboration risk of bias tool and the effective public health practice project quality assessment tool: Methodological research. Journal of Evaluation in Clinical Practice, 18(1), 12–18. 10.1111/j.1365-2753.2010.01516.x 20698919

[jcv212300-bib-0003] Barnett, R. , & Hunter, M. (2012). Adjustment of siblings of children with mental health problems: Behaviour, self‐concept, quality of life and family functioning. Journal of Child and Family Studies, 21(2), 262–272. 10.1007/s10826-011-9471-2

[jcv212300-bib-0004] Bowen, M. (1978). Family therapy in clinical practice. Jason Aronson.

[jcv212300-bib-0005] Deeks, J. J. , Dinnes, J. , D’Amico, R. , Sowden, A. J. , Sakarovitch, C. , Song, F. , Petticrew, M. , & Altman, D. , & & European Carotid Surgery Trial Collaborative Group . (2003). Evaluating non‐randomised intervention studies. Health Technology Assessment, 7(27), iii–173. 10.3310/hta7270 14499048

[jcv212300-bib-0006] Faulconbridge, J. , Law, D. , & Laffan, A. (2015). What good looks like in psychological services for children, young people and their families, the child & family clinical psychology review. British Psychological Society.

[jcv212300-bib-0007] Feriante, J. , Shayani, A. , Lauer, E. , Pressman, A. , & Rubin, E. (2022). Sibling support program: A novel peer support intervention for parents, caregivers and siblings of youth experiencing mental illness. Healthcare, 10(5), 908. 10.3390/healthcare10050908 35628046 PMC9140975

[jcv212300-bib-0008] Foster, K. , McPhee, I. , Fethney, J. , & McCloughen, A. (2016). Outcomes of the ON FIRE peer support programme for children and adolescents in families with mental health problems. Child & Family Social Work, 21(3), 295–306. 10.1111/cfs.12143

[jcv212300-bib-0009] Goodman, R. (2001). Psychometric properties of the strengths and difficulties questionnaire. Journal of the American Academy of Child & Adolescent Psychiatry, 40(11), 1337–1345. 10.1097/00004583-200111000-00015 11699809

[jcv212300-bib-0010] Goodyear, M. , Cuff, R. , Maybery, D. , & Reupert, A. (2009). Champs: A peer support program for children of parents with a mental illness. Australian E‐Journal for the Advancement of Mental Health, 8(3), 296–304. 10.5172/jamh.8.3.296

[jcv212300-bib-0011] Hartling, L. , Milne, A. , Tjosvold, L. , Wrightson, D. , Gallivan, J. , & Newton, A. S. (2014). A systematic review of interventions to support siblings of children with chronic illness or disability. Journal of Paediatrics and Child Health, 50(10), E26–E38. 10.1111/j.1440-1754.2010.01771.x 20598075

[jcv212300-bib-0012] Hoffmann, T. C. , Glasziou, P. P. , Boutron, I. , Milne, R. , Perera, R. , Moher, D. , Altman, D. G. , Barbour, V. , Macdonald, H. , Johnston, M. , Lamb, S. E. , Dixon‐Woods, M. , McCulloch, P. , Wyatt, J. C. , Chan, A. W. , & Michie, S. (2014). Better reporting of interventions: Template for intervention description and replication (TIDieR) checklist and guide. BMJ, 348(mar07 3), g1687. 10.1136/bmj.g1687 24609605

[jcv212300-bib-0013] Kirchhofer, S. M. , Orm, S. , Haukeland, Y. B. , Fredriksen, T. , Wakefield, C. E. , & Fjermestad, K. W. (2022). A systematic review of social support for siblings of children with neurodevelopmental disorders. Research in Developmental Disabilities, 126, 104234. 10.1016/j.ridd.2022.104234 35468570

[jcv212300-bib-0014] Kozlowska, K. , & Elliott, B. (2017). Don’t forget the siblings: School‐aged siblings of children presenting to mental health services show at‐risk patterns of attachment. Clinical Child Psychology and Psychiatry, 22(2), 245–259. 10.1177/1359104516653993 27324573

[jcv212300-bib-0015] Laurent, J. , Catanzaro, S. J. , Joiner Jr, T. E. , Rudolph, K. D. , Potter, K. I. , Lambert, S. , Osborne, L. , & Gathright, T. (1999). A measure of positive and negative affect for children: Scale development and preliminary validation. Psychological Assessment, 11(3), 326–338. 10.1037/1040-3590.11.3.326

[jcv212300-bib-0016] Lawson, D. W. , & Mace, R. (2010). Siblings and childhood mental health: Evidence for a later‐born advantage. Social Science & Medicine, 70(12), 2061–2069. 10.1016/j.socscimed.2010.03.009 20381935

[jcv212300-bib-0017] Lock, J. , & Le Grange, D. (2013). Treatment manual for anorexia nervosa: A family‐based approach (2nd ed.). Guilford Press.

[jcv212300-bib-0019] Long, H. A. , French, D. P. , & Brooks, J. M. (2020). Optimising the value of the critical appraisal skills programme (CASP) tool for quality appraisal in qualitative evidence synthesis. Research Methods in Medicine & Health Sciences, 1(1), 31–42. 10.1177/2632084320947559

[jcv212300-bib-0020] Ma, N. , Furber, G. , Roberts, R. , & Winefield, H. (2016). Caregiver perceptions of mental health problems and treatment utilisation in siblings of children with mental health problems. Journal of Mental Health, 25(2), 165–168. 10.3109/09638237.2015.1101413 26617080

[jcv212300-bib-0021] Ma, N. , Roberts, R. , Winefield, H. , & Furber, G. (2017). The quality of family relationships for siblings of children with mental health problems: A 20‐year systematic review. Journal of Family Studies, 23(3), 309–332. 10.1080/13229400.2015.1108994

[jcv212300-bib-0022] Ma, N. , Roberts, R. , Winefield, H. , & Furber, G. (2020). A dimensional approach to the mental health of siblings of children with mental health problems: A 20‐year systematic review. Journal of Family Studies, 26(2), 308–328. 10.1080/13229400.2017.1375966

[jcv212300-bib-0023] Marsh, D. T. , & Johnson, D. L. (1997). The family experience of mental illness: Implications for intervention. Professional Psychology: Research and Practice, 28(3), 229–237. 10.1037/0735-7028.28.3.229

[jcv212300-bib-0024] McGrath, L. , Wilson, C. E. , & Buckmaster, A. (2024). ‘No one else understands’, ‘I wouldn’t want to pity myself over something that’s not really my problem’: Siblings’ experiences of their adolescent brothers and sisters’ inpatient treatment for mental health difficulties. Child and Adolescent Mental Health, 29(1), 4–13. 10.1111/camh.12645 36846899

[jcv212300-bib-0025] McKenzie Smith, M. , Pinto Pereira, S. , Chan, L. , Rose, C. , & Shafran, R. (2018). Impact of well‐being interventions for siblings of children and young people with a chronic physical or mental health condition: A systematic review and meta‐analysis. Clinical Child and Family Psychology Review, 21(2), 246–265. 10.1007/s10567-018-0253-x 29450764 PMC5899110

[jcv212300-bib-0026] McManus, S. , Gunnell, D. , Cooper, C. , Bebbington, P. E. , Howard, L. M. , Brugha, T. , Jenkins, R. , Hassiotis, A. , Weich, S. , & Appleby, L. (2019). Prevalence of non‐suicidal self‐harm and service contact in England, 2000–14: Repeated cross‐sectional surveys of the general population. The Lancet Psychiatry, 6(7), 573–581. 10.1016/S2215-0366(19)30188-9 31175059 PMC7646286

[jcv212300-bib-0027] Meyer, D. J. , & Vadasy, P. F. (2008). Sibshops: Workshops for siblings of children with special needs. Brookes Publishing Company.

[jcv212300-bib-0028] Moher, D. , Liberati, A. , Tetzlaff, J. , & Altman, D. G. , & Prisma Group . (2010). Preferred reporting items for systematic reviews and meta‐analyses: The PRISMA statement. International Journal of Surgery, 8(5), 336–341. 10.1016/j.ijsu.2010.02.007 20171303

[jcv212300-bib-0029] Perrin, E. C. , Newacheck, P. , Pless, I. B. , Drotar, D. , Gortmaker, S. L. , Leventhal, J. , Perrin, J. M. , Stein, R. E. , Walker, D. K. , & Weitzman, M. (1993). Issues involved in the definition and classification of chronic health conditions. Pediatrics, 91(4), 787–793. 10.1542/peds.91.4.787 8464668

[jcv212300-bib-0030] Popay, J. , Roberts, H. , Sowden, A. , Petticrew, M. , Arai, L. , Rodgers, M. , Britten, N. , Roen, K. , & Duffy, S. (2006). Guidance on the conduct of narrative synthesis in systematic reviews. A Product from the ESRC Methods Programme Version, 1(1), b92.

[jcv212300-bib-0031] Rubin, E. , Ostrowsky, L. , Janopaul‐Naylor, E. , Sehgal, P. , Cama, S. , Tanski, E. , & Curtin, C. (2018). The sibling support demonstration project: A pilot study assessing feasibility, preliminary effectiveness, and participant satisfaction. Adolescent Psychiatry, 8(1), 48–60. 10.2174/2210676608666180208160524

[jcv212300-bib-0032] Sadler, K. , Vizard, T. , Ford, T. , Goodman, A. , Goodman, R. , & McManus, S. (2018). Mental health of children and young people in England, 2017: Trends and characteristics [report]. NHS Digital. Retrieved from https://digital.nhs.uk/data‐and‐information/publications/statistical/mental‐health‐of‐children‐and‐young‐people‐in‐england/2017/2017

[jcv212300-bib-0033] Sangha, S. , Anderson, J. K. , & Burn, A.‐M. (2023). A qualitative study investigating the experiences of young adults caring for a sibling with disability within immigrant families in the UK: “Challenges are just the constant.”. Journal of Intellectual & Developmental Disability, 48(4), 1–11. 10.3109/13668250.2023.2233238 39815882

[jcv212300-bib-0034] Shivers, C. , Russon, J. , Benson, M. J. , King, A. , & Textoris, S. (2022). Siblings’ role positions and perceptions of mental illness. Contemporary Family Therapy, 45(3), 1–11. 10.1007/s10591-022-09637-6

[jcv212300-bib-0035] Snyder, C. R. , Hoza, B. , Pelham, W. E. , Rapoff, M. , Ware, L. , Danovsky, M. , Highberger, L. , Ribinstein, H. , & Stahl, K. J. (1997). The development and validation of the Children’s Hope Scale. Journal of Pediatric Psychology, 22(3), 399–421. 10.1093/jpepsy/22.3.399 9212556

[jcv212300-bib-0036] Solmi, M. , Radua, J. , Olivola, M. , Croce, E. , Soardo, L. , Salazar de Pablo, G. , Il Shin, J. , Kirkbride, J. B. , Jones, P. , Kim, J. H. , Carvalho, A. F. , Seeman, M. V. , Correll, C. U. , & Fusar‐Poli, P. (2022). Age at onset of mental disorders worldwide: Large‐scale meta‐analysis of 192 epidemiological studies. Molecular Psychiatry, 27(1), 281–295. 10.1038/s41380-021-01161-7 34079068 PMC8960395

[jcv212300-bib-0037] Stålberg, G. , Ekerwald, H. , & Hultman, C. M. (2004). Siblings of patients with schizophrenia: Sibling bond, coping patterns, and fear of possible schizophrenia heredity. Schizophrenia Bulletin, 30(2), 445–458. 10.1093/oxfordjournals.schbul.a007091 15279059

[jcv212300-bib-0038] Stein, D. J. , Szatmari, P. , Gaebel, W. , Berk, M. , Vieta, E. , Maj, M. , De Vries, Y. A. , Roest, A. M. , De Jonge, P. , Maercker, A. , Brewin, C. R. , Pike, K. M. , Grilo, C. M. , Fineberg, N. A. , Briken, P. , Cohen‐Kettenis, P. T. , & Reed, G. M. (2020). Mental, behavioral and neurodevelopmental disorders in the ICD‐11: An international perspective on key changes and controversies. BMC Medicine, 18(1), 1–24. 10.1186/s12916-020-1495-2 31983345 PMC6983973

[jcv212300-bib-0039] Taylor, J. L. , Greenberg, J. S. , Seltzer, M. M. , & Floyd, F. J. (2008). Siblings of adults with mild intellectual deficits or mental illness: Differential life course outcomes. Journal of Family Psychology, 22(6), 905–914. 10.1037/a0012603 19102611 PMC2610343

[jcv212300-bib-0040] Thomas, S. , Reddy, N. K. , & Sagar, K. J. V. (2016). Review on psychosocial interventions for siblings of children with autism spectrum disorder (ASD). Journal of Psychosocial Rehabilitation and Mental Health, 3(2), 101–107. 10.1007/s40737-016-0064-7

[jcv212300-bib-0041] Tudor, M. E. , & Lerner, M. D. (2015). Intervention and support for siblings of youth with developmental disabilities: A systematic review. Clinical Child and Family Psychology Review, 18, 1–23. 10.1007/s10567-014-0175-1 25315924

[jcv212300-bib-0042] V an der Sanden, R. L. , Pryor, J. B. , Stutterheim, S. E. , Kok, G. , & Bos, A. E. (2016). Stigma by association and family burden among family members of people with mental illness: The mediating role of coping. Social Psychiatry and Psychiatric Epidemiology, 51(9), 1233–1245. 10.1007/s00127-016-1256-x 27357819 PMC5025495

[jcv212300-bib-0043] Van Langenberg, T. , Duncan, R. E. , Allen, J. S. , Sawyer, S. M. , Le Grange, D. , & Hughes, E. K. (2018). “They don’t really get heard”: A qualitative study of sibling involvement across two forms of family‐based treatment for adolescent anorexia nervosa. Eating Disorders, 26(4), 373–387. 10.1080/10640266.2018.1453632 29683775

[jcv212300-bib-0044] V an Langenberg, T. , Sawyer, S. M. , Le Grange, D. , & Hughes, E. K. (2016). Psychosocial well‐being of siblings of adolescents with anorexia nervosa. European Eating Disorders Review, 24(6), 438–445. 10.1002/erv.2469 27501269

[jcv212300-bib-0045] Waffenschmidt, S. , Knelangen, M. , Sieben, W. , Bühn, S. , & Pieper, D. (2019). Single screening versus conventional double screening for study selection in systematic reviews: A methodological systematic review. BMC Medical Research Methodology, 19(1), 1–9. 10.1186/s12874-019-0782-0 31253092 PMC6599339

[jcv212300-bib-0046] Wind, G. , & Jorgensen, G. (2019). “It has been fun. Super‐duper fantastic”: Findings from a Danish respite programme to support young carers. Health and Social Care in the Community, 28(1), 100–109. 10.1111/hsc.12844 31476094

[jcv212300-bib-0047] Wolfe, B. , Song, J. , Greenberg, J. S. , & Mailick, M. R. (2014). Ripple effects of developmental disabilities and mental illness on nondisabled adult siblings. Social Science & Medicine, 108, 1–9. 10.1016/j.socscimed.2014.01.021 24607704 PMC4079586

[jcv212300-bib-0048] Wolff, B. , Magiati, I. , Roberts, R. , Pellicano, E. , & Glasson, E. J. (2022). Risk and resilience factors impacting the mental health and wellbeing of siblings of individuals with neurodevelopmental conditions: A mixed methods systematic review. Clinical Psychology Review, 98, 102217. 10.1016/j.cpr.2022.102217 36368218

[jcv212300-bib-0049] Wolff, B. , Magiati, I. , Roberts, R. , Skoss, R. , & Glasson, E. J. (2023). Psychosocial interventions and support groups for siblings of individuals with neurodevelopmental conditions: A mixed methods systematic review of sibling self‐reported mental health and wellbeing outcomes. Clinical Child and Family Psychology Review, 26(1), 143–189. 10.1007/s10567-022-00413-4 36175605 PMC9879846

