# Supplementary material for: Interventions for siblings of children and young people with mental health conditions: A systematic review
Source: JCPP Adv. 2025 Jan 23;5(2):e12300. doi: 10.1002/jcv2.12300 (PMC12159328; doi:10.1002/jcv2.12300)
Supplement: Supplementary file 1 — Supplementary material [file JCV2-5-e12300-s001.docx]

**Supplementary Table 1**

*Embase search strategy*

| 13 | limit 12 to english language |
| --- | --- |
| 12 | 10 and 1980:2023.(sa_year). |
| 11 | 10 and 1980:2023.(sa_year). |
| 10 | 1 and 7 and 8 and 9 |
| 9 | 2 or 3 or 4 or 5 or 6 |
| 8 | Sibling/ or (Sibling* or brother* or sister* or half-sibling* or half-brother* or half-sister* or step-sibling* or step-brother* or step-sister*).ti,ab. |
| 7 | "Intervention"/ or (intervention* or programme* or program* or training or tool* or support).ti,ab. |
| 6 | "Mental Health"/ or (((psychol* or psychiatr* or mental or emotional or behavioral or behavioural) adj2 (condition or problem or health or disorder or disease or ill or wellbeing)) or psychiatr*).ti,ab. |
| 5 | "Eating Disorders"/ or (((feeding or eating or "binge eating" or "binge-eating") adj2 disorder*) or (atypical and (bulimia or anorexia)) or (anorexia and bulimia and nervosa) or ARFID or ED or binging or "disordered eating" or neophobia or anorexi* or bulimi* or "anorexia nervosa" or "bulimia nervosa" or "eating disorder" or "eating disorder not otherwise specified" or "binge-eating disorder*" or "binge eat").ti,ab. |
| 4 | anxiety/ or (anx* or panic or phobi* or "affective disorder*" or "mood disorder*").ti,ab. |
| 3 | depression/ or (depress* or dysthymi* or MDD or "seasonal affective disorder*" or anhedonia or dysphoria).ti,ab. |
| 2 | self-harm/ or ("self harm*" or "self-harm*" or "self injur*" or "self-injur*" or "non-suicidal-self-injur*" or "NSSI" or "DSH" or "self-mutilation" or self-poisoning or "head banging" or "self-inflicted wound*" or "deliberate self-harm").ti,ab. |
| 1 | exp youth/ or ((young and (people or person*)) or child* or adolescent* or teen*).ti,ab. |

**Supplementary Table 2**

*Medline search strategy*

| S8 AND (S3 OR S4 OR S5 OR S6 OR S7) AND S2 AND S1 | Limiters - Publication Year: 1980-; Publication Type: Journal Article; English  Expanders - Apply equivalent subjects  Search modes - Boolean/Phrase |
| --- | --- |
| S8 | young people/ OR ((young and (people or person*)) or child* or adolescent* or teen*).ti,ab. |
| S7 | self-harm/ OR ("self harm*" or "self-harm*" OR "self injur*" or "self-injur*" or "non-suicidal-self-injur*" or "NSSI" or "DSH" or "self-mutilation" or self-poisoning or "head banging" or "self-inflicted wound*" or "deliberate self-harm").ti,ab. |
| S6 | depression/ OR (depress* or dysthymi* or MDD or "seasonal affective disorder*" or anhedonia or dysphoria).ti,ab. |
| S5 | anxiety/ OR (anx* or panic or phobi* or "affective disorder*" or "mood disorder*").ti,ab. |
| S4 | "Eating Disorders"/ OR (((feeding or eating or "binge eating" or "binge-eating") adj1 disorder*) or (atypical and (bulimia or anorexia)) or ((anorexia and bulimia) AND nervosa) OR ARFID or ED or binging or "disordered eating" or neophobia or anorexi* or bulimi* or "anorexia nervosa" or "bulimia nervosa" or "eating disorder" or "eating disorder not otherwise specified" or "binge-eating disorder*" or "binge eat").ti,ab. |
| S3 | "Mental Health"/ OR ((psychol* or psychiatr* or mental or emotional or behavioral or behavioural) adj1 (condition or problem or health or disorder or disease or ill or wellbeing) OR psychiatr*).ti,ab. |
| S2 | "Intervention"/ OR (intervention* or programme* or program* or training or tool* or support).ti,ab. |
| S1 | Sibling/ OR (Sibling* or brother* or sister* or half-sibling* or half-brother* or half-sister* or step-sibling* or step-brother* or step-sister*).ti,ab. |

**Supplementary Table 3**

*ASSIA search strategy*

| S8 AND (S3 OR S4 OR S5 OR S6 OR S7) AND S2 AND S1 | Limiters - Publication Year: 1980-; Publication Type: Peer Reviewed Journal; English; Scholarly Journals  Expanders - Apply equivalent subjects  Search modes - Boolean/Phrase |
| --- | --- |
| S8 | SU("YOUNG PEOPLE") OR title((young AND (people OR person*)) OR child* OR adolescent* OR teen*) OR abstract((young AND (people OR person*)) OR child* OR adolescent* OR teen*) |
| S7 | SU(“self harm”) OR title("self harm*" or "self-harm*" OR "self injur*" or "self-injur*" or "non-suicidal-self-injur*" or "NSSI" or "DSH" or "self-mutilation" or self-poisoning or "head banging" or "self-inflicted wound*" or "deliberate self-harm")  OR abstract ("self harm*" or "self-harm*" OR "self injur*" or "self-injur*" or "non-suicidal-self-injur*" or "NSSI" or "DSH" or "self-mutilation" or self-poisoning or "head banging" or "self-inflicted wound*" or "deliberate self-harm") |
| S6 | SU (“Depression”) OR title(depress* or dysthymi* or "MDD" or "seasonal affective disorder" OR anhedonia" or dysphoria) OR abstract(depress* or dysthymi* or "MDD" or "seasonal affective disorder" OR anhedonia" or dysphoria) |
| S5 | SU( "Anxiety") OR title("Anx*" OR panic OR "phobi*" OR “affective disorder*” OR "mood disorder*" OR anxiety*) OR abstract (“Anx*” OR panic OR "phobi*" OR "affective disorder*" OR "mood disorder*" OR anxiety*) |
| S4 | SU("Eating Disorders")  OR title(((feeding or eating or "binge eating" or "binge-eating") NEAR/1 disorder*) or (atypical and (bulimia or anorexia)) or ((anorexia and bulimia) AND nervosa) OR ARFID or ED or binging or "disordered eating" or neophobia or anorexi* or bulimi* or "anorexia nervosa" or "bulimia nervosa" or "eating disorder" or "eating disorder not otherwise specified" or "binge-eating disorder*" or "binge eat")  OR abstract(((feeding or eating or "binge eating" or "binge-eating") NEAR/1 disorder*) or (atypical and (bulimia or anorexia)) or ((anorexia and bulimia) AND nervosa) OR ARFID or ED or binging or "disordered eating" or neophobia or anorexi* or bulimi* or "anorexia nervosa" or "bulimia nervosa" or "eating disorder" or "eating disorder not otherwise specified" or "binge-eating disorder*" or "binge eat") |
| S3 | SU("Mental Health") OR abstract ((psychol* or psychiatr* or mental or emotional or behavioral or behavioural) NEAR/1 (condition or problem or health or disorder or disease or ill or wellbeing) OR psychiatry*)  OR Title(((psychol* or psychiatr* or mental or emotional or behavioral or behavioural) NEAR/1 (condition or problem or health or disorder or disease or ill or wellbeing) OR psychiatry*)) |
| S2 | SU(“Intervention") OR abstract(intervention* or programme* or program* or training or tool* or support) OR Title(intervention* or programme* or program* or training or tool* or support) |
| S1 | SU(“ Sibling”) OR Abstract(Sibling* or brother* or sister* or half-sibling* or half-brother* or half-sister* or step-sibling* or step-brother* or step-sister*) OR Title( Sibling* or brother* or sister* or half-sibling* or half-brother* or half-sister* or step-sibling* or step-brother* or step-sister*) |

**Supplementary Table 4**

*ERIC search strategy*

| S8 AND (S3 OR S4 OR S5 OR S6 OR S7) AND S2 AND S1 | Limiters - Publication Year: 1980-; Publication Type: Peer Reviewed Journal; English  Expanders - Apply equivalent subjects  Search modes - Boolean/Phrase |
| --- | --- |
| S8 | DE("youth") OR  TI((young AND (people OR person*)) OR child* OR adolescent* OR teen*) OR AB((young AND (people OR person*)) OR child* OR adolescent* OR teen*)) |
| S7 | DE(“self harm”) OR TI("self harm*" or "self-harm*" OR "self injur*" or "self-injur*" or "non-suicidal-self-injur*" or "NSSI" or "DSH" or "self-mutilation" or self-poisoning or "head banging" or "self-inflicted wound*" or "deliberate self-harm")  OR AB("self harm*" or "self-harm*" OR "self injur*" or "self-injur*" or "non-suicidal-self-injur*" or "NSSI" or "DSH" or "self-mutilation" or self-poisoning or "head banging" or "self-inflicted wound*" or "deliberate self-harm") |
| S6 | DE(“Depression”) OR TI(depress* or dysthymi* or "MDD" or "seasonal affective disorder" OR anhedonia" or dysphoria) OR AB(depress* or dysthymi* or "MDD" or "seasonal affective disorder" OR anhedonia" or dysphoria) |
| S5 | DE"(Anxiety") OR TI("Anx*" OR panic OR "phobi*" OR “affective disorder*” OR "mood disorder*" OR anxiety*) OR AB (“Anx*” OR panic OR "phobi*" OR "affective disorder*" OR "mood disorder*" OR anxiety*) |
| S4 | DE("Eating Disorders")  OR TI(((feeding or eating or "binge eating" or "binge-eating") N1 disorder*) or (atypical and (bulimia or anorexia)) or ((anorexia and bulimia) AND nervosa) OR ARFID or ED or binging or "disordered eating" or neophobia or anorexi* or bulimi* or "anorexia nervosa" or "bulimia nervosa" or "eating disorder" or "eating disorder not otherwise specified" or "binge-eating disorder*" or "binge eat")  OR AB(((feeding or eating or "binge eating" or "binge-eating") N1 disorder*) or (atypical and (bulimia or anorexia)) or ((anorexia and bulimia) AND nervosa) OR ARFID or ED or binging or "disordered eating" or neophobia or anorexi* or bulimi* or "anorexia nervosa" or "bulimia nervosa" or "eating disorder" or "eating disorder not otherwise specified" or "binge-eating disorder*" or "binge eat") |
| S3 | DE("Mental Health") OR AB((psychol* or psychiatr* or mental or emotional or behavioral or behavioural) N1 (condition or problem or health or disorder or disease or ill or wellbeing) OR psychiatry*)  OR TI(((psychol* or psychiatr* or mental or emotional or behavioral or behavioural) N1 (condition or problem or health or disorder or disease or ill or wellbeing) OR psychiatry*)) |
| S2 | DE(“Intervention") OR AB(intervention* or programme* or program* or training or tool* or support) OR TI(intervention* or programme* or program* or training or tool* or support) |
| S1 | DE(“ Sibling”) OR AB(Sibling* or brother* or sister* or half-sibling* or half-brother* or half-sister* or step-sibling* or step-brother* or step-sister*) OR TI( Sibling* or brother* or sister* or half-sibling* or half-brother* or half-sister* or step-sibling* or step-brother* or step-sister*) |

**Supplementary Table 5**

*British Education Index search strategy*

| S8 AND (S3 OR S4 OR S5 OR S6 OR S7) AND S2 AND S1 | Limiters - Publication Year: 1980-; Publication Type: Peer Reviewed Journal; English  Expanders - Apply equivalent subjects  Search modes - Boolean/Phrase |
| --- | --- |
| S8 | (MH “young people”) OR (young and (people or person*)) or child* or adolescent* or teen*).ti,ab. |
| S7 | (MH“self-harm") OR ("self harm*" or "self-harm*" OR "self injur*" or "self-injur*" or "non-suicidal-self-injur*" or "NSSI" or "DSH" or "self-mutilation" or self-poisoning or "head banging" or "self-inflicted wound*" or "deliberate self-harm")).ti,ab. |
| S6 | (MH “Depression”) OR (depress* or dysthymi* or "MDD" or "seasonal affective disorder" OR anhedonia" or dysphoria).ti,ab. |
| S5 | (MH “Anxiety”) OR (“Anx*” OR panic OR “*phobi” OR “phobi*” OR “affective disorder*” OR “mood disorder*” OR * anxiety*).ti,ab. |
| S4 | (MH“Eating Disorders") OR AB (((feeding or eating or "binge eating" or "binge-eating“ N1 (disorder*)) or (atypical and (bulimia or anorexia)) or ((anorexia and bulimia) AND nervosa OR “ARFID” or” ED” or binging or "disordered eating" or neophobia or anorexi* or bulimi* or "anorexia nervosa" or "bulimia nervosa" or "eating disorder" or "eating disorder not otherwise specified" or "binge-eating disorder*" or "binge eat").ti,ab. |
| S3 | (MH"Mental Health"/ OR ((psychol* or psychiatr* or mental or emotional or behavioral or behavioural) n1 (condition or problem or health or disorder or disease or ill or wellbeing) OR psychiatr*).ti,ab. |
| S2 | (MH"Intervention") OR (intervention* or programme* or program* or training or tool* or support).ti,ab, |
| S1 | (MH "Sibling") OR (Sibling* or brother* or sister* or half-sibling* or half-brother* or half-sister* or step-sibling* or step-brother* or step-sister*).ti,ab. |

**Supplementary Table 6**

*Child and Developmental and Adolescent Studies*

| S8 AND (S3 OR S4 OR S5 OR S6 OR S7) AND S2 AND S1 | Limiters - Publication Year: 1980-; Publication Type: Peer Reviewed Journal; English  Expanders - Apply equivalent subjects  Search modes - Boolean/Phrase |
| --- | --- |
| S8 | (MH “young people”) OR (young and (people or person*)) or child* or adolescent* or teen*).ti,ab. |
| S7 | (MH“self-harm") OR ("self harm*" or "self-harm*" OR "self injur*" or "self-injur*" or "non-suicidal-self-injur*" or "NSSI" or "DSH" or "self-mutilation" or self-poisoning or "head banging" or "self-inflicted wound*" or "deliberate self-harm")).ti,ab. |
| S6 | (MH “Depression”) OR (depress* or dysthymi* or "MDD" or "seasonal affective disorder" OR anhedonia" or dysphoria).ti,ab. |
| S5 | (MH “Anxiety”) OR (“Anx*” OR panic OR “*phobi” OR “phobi*” OR “affective disorder*” OR “mood disorder*” OR * anxiety*).ti,ab. |
| S4 | (MH“Eating Disorders") OR AB (((feeding or eating or "binge eating" or "binge-eating“ N1 (disorder*)) or (atypical and (bulimia or anorexia)) or ((anorexia and bulimia) AND nervosa OR “ARFID” or” ED” or binging or "disordered eating" or neophobia or anorexi* or bulimi* or "anorexia nervosa" or "bulimia nervosa" or "eating disorder" or "eating disorder not otherwise specified" or "binge-eating disorder*" or "binge eat").ti,ab. |
| S3 | (MH"Mental Health"/ OR ((psychol* or psychiatr* or mental or emotional or behavioral or behavioural) n1 (condition or problem or health or disorder or disease or ill or wellbeing) OR psychiatr*).ti,ab. |
| S2 | (MH"Intervention") OR (intervention* or programme* or program* or training or tool* or support).ti,ab, |
| S1 | (MH "Sibling") OR (Sibling* or brother* or sister* or half-sibling* or half-brother* or half-sister* or step-sibling* or step-brother* or step-sister*).ti,ab. |

**Supplementary Table 7**

*Psycinfo*

| S9 | S8 AND (S3 OR S4 OR S5 OR S6 OR S7) AND S2 AND S1 | Limiters - Publication Year: 1980-; Publication Type: Peer Reviewed Journal; English  Expanders - Apply equivalent subjects  Search modes - Boolean/Phrase |
| --- | --- | --- |
|  | S8 | DE ("young people") OR AB (young and (people or person*)) or child* or adolescent* or teen*) |
|  | S7 | DE ("self-harm") OR AB ("self harm*" or "self-harm*" OR "self injur*" or "self-injur*" or "non-suicidal-self-injur*" or "NSSI" or "DSH" or "self-mutilation" or self-poisoning or "head banging" or "self-inflicted wound*" or "deliberate self-harm")) |
|  | S6 | DE ("Depression") OR AB(depress* or dysthymi* or "MDD" or "seasonal affective disorder" OR anhedonia" or dysphoria) |
|  | S5 | DE ("Anxiety") OR AB(“Anx*” OR panic OR “*phobi” OR “phobi*” OR “affective disorder*” OR “mood disorder*” OR * anxiety*) |
|  | S4 | DE ("Eating Disorders") OR AB(((feeding or eating or "binge eating" or "binge-eating“ N2 (disorder*)) or (atypical and (bulimia or anorexia)) or ((anorexia and bulimia) AND nervosa OR ARFID or ED or binging or "disordered eating" or neophobia or anorexi* or bulimi* or "anorexia nervosa" or "bulimia nervosa" or "eating disorder" or "eating disorder not otherwise specified" or "binge-eating disorder*" or "binge eat") |
|  | S3 | DE ("Mental Health") OR AB ((psychol* or psychiatr* or mental or emotional or behavioral or behavioural) n1 (condition or problem or health or disorder or disease or ill or wellbeing) OR psychiatr*) |
|  | S2 | DE ("Intervention") OR AB (intervention* or programme* or program* or training or tool* or support) |
|  | S1 | DE ("Sibling") OR AB (Sibling* or brother* or sister* or half-sibling* or half-brother* or half-sister* or step-sibling* or step-brother* or step-sister*) |

**Supplementary Figure 1**

*Quality appraisal scores*

|  |  | Author | | |
| --- | --- | --- | --- | --- |
|  |  | **Van Langenberg 2016** | Rubin 2018 | Feriante 2022 |
| **Quality criteria** | Selection Bias | **Weak** | Weak | Weak |
|  | Study Design | **Moderate** | Weak | Weak |
|  | Confounders | **N/A** | N/A | Weak |
|  | Blinding | **Weak** | Weak | Weak |
|  | Data Collection Method | **Weak** | Weak | Weak |
|  | Withdrawals and Dropouts | **Moderate** | Strong | N/A |
|  | Global Ratings | **Weak** | Weak | Weak |

*Note: Quantitative results that met our inclusion criteria are presented in bold.*

**Supplementary Table 8**

*Data extraction table*

| First author, year | Intervention | Sample size and demographic characteristics | Outcome measures (indicate measures used and whether self-, parent-, carer- or clinician-reported) | 1. Mental health/well-being/ quality of life | 2. Attitudes/ perceived effectiveness/ acceptability | Comments |
| --- | --- | --- | --- | --- | --- | --- |

**Supplementary Table 9**

*Data extraction table*

| First author, year | Rationale/ theory of change | What | Who provided | How | Where | When and how much | Tailoring | Modifications | How well |
| --- | --- | --- | --- | --- | --- | --- | --- | --- | --- |

**Supplementary Table 10**

*Near-miss study*

| First author, year | Intervention | Sample size and demographic characteristics | Outcome measures (indicate measures used and whether self-, parent-, carer- or clinician-reported) | 1. Mental health/well-being/ quality of life | 2. Attitudes/ perceived effectiveness/ acceptability | Comments |
| --- | --- | --- | --- | --- | --- | --- |
| Foster, 2016 | on FIRE peer support program | -n=64 CYP  -age 8-17 (M = 11.7, SD = 2.5); 54.7% female - 11 (19%) CYP had sibling MHC  - ill relative: type of MHC (by order of prevalence): depression, anxiety, bipolar, BPD, psychosis, PTSD, OCD, panic disorder, CD, social anxiety, schizoaffective disorder, adjustment disorder | -SDQ pre- and post- intervention, CYP-rated (at ages 13-17) and parent-rated (at all ages) -suite of child (ages 8-12) or adolescent (13-17) measures -Children's Hope Scale (CHS; Snyder et al., 1997; all ages) -Kids Coping Scale (KCS; Goodyear et al., 2009; all ages) -Positive and Negative Affect Scale for Children (PANAS-C; Laurent et al., 1999; all ages) | -Greater SDQ scores parent-reported compared to norm population.  -No sign. changes in self-or parent-reported SDQ scales  -PANAS no sign. change.  -CHS & KCS sign. Increase. | -54.8% of caregivers indicated no change in CYP problems, 43% reported an improvement -81.8% of CYP indicated improvement of their problems, 18.2% reported no change -94.9% of participants found programme enjoyable  -92.3% would recommend programme to other CYP -participant mean rating of programme: 8.5/10, SD=1.7 | -Low reliability for KCS scale with and outside family connection.  -SDQ measures: no Bonferroni corrections performed. |

Note: BPD: borderline personality disorder, PTSD: post-traumatic stress disorder, OCD: obsessive-compulsive disorder, CD: conduct disorder, sign.: significant
